# Pen-Based Swine Oral Fluid Samples Contain Both Environmental and Pig-Derived Targets

**DOI:** 10.3390/ani14050766

**Published:** 2024-02-29

**Authors:** Grzegorz Tarasiuk, Marta D. Remmenga, Kathleen C. O’Hara, Marian K. Talbert, Marisa L. Rotolo, Pam Zaabel, Danyang Zhang, Luis G. Giménez-Lirola, Jeffrey J. Zimmerman

**Affiliations:** 1 Department of Veterinary Diagnostic and Production Animal Medicine, College of Veterinary Medicine, Iowa State University, Ames, IA 50011, USA; tarasiuk@iastate.edu (G.T.);; 2 USDA: VS: Strategy and Policy, Center for Epidemiology and Animal Health, Fort Collins, CO 80526, USA; 3 PIC North America, Hendersonville, TN 37075, USA; 4 National Pork Board, Des Moines, IA 50325, USA; 5 Department of Statistics, College of Liberal Arts and Sciences, Iowa State University, Ames, IA 50010, USA

**Keywords:** surveillance, oral fluids, sampling, tracer, environmental sampling, swine

## Abstract

**Simple Summary:**

The transfer of a target directly from pigs or indirectly from the environment into pen-based oral fluid samples was documented using a fluorescent tracer (red food coloring). Pens of ~30, ~60, and ~125 14-week-old pigs (32 pens/size) on commercial swine farms received one of two treatments: (1) pig exposure, i.e., ~3.5 mL of tracer solution sprayed into the mouth of 10% of the pigs in the pen; (2) environmental exposure, i.e., 20 mL of tracer solution was poured on the floor in the center of the pen. Oral fluids collected one day prior to treatment (baseline fluorescence control) and immediately after treatment were tested for fluorescence. Data were evaluated by receiver operating characteristic (ROC) analysis, with Youden’s J statistic used to set a threshold. Pretreatment oral fluid samples with fluorescence responses above the ROC threshold were removed from further analysis (7 of 96 samples). Analysis of post-treatment samples showed detectable levels of red food coloring in oral fluid samples from 78 of 89 pens (87.6%), including 43 of 47 (91.5%) pens in which pigs were directly exposed and 35 of 42 (83.3%) pens in which the tracer was placed in the environment. Thus, oral fluid samples contain both pig-derived and environmental targets.

**Abstract:**

Laboratory methods for detecting specific pathogens in oral fluids are widely reported, but there is little research on the oral fluid sampling process itself. In this study, a fluorescent tracer (diluted red food coloring) was used to test the transfer of a target directly from pigs or indirectly from the environment to pen-based oral fluid samples. Pens of ~30, ~60, and ~125 14-week-old pigs (32 pens/size) on commercial swine farms received one of two treatments: (1) pig exposure, i.e., ~3.5 mL of tracer solution sprayed into the mouth of 10% of the pigs in the pen; (2) environmental exposure, i.e., 20 mL of tracer solution was poured on the floor in the center of the pen. Oral fluids collected one day prior to treatment (baseline fluorescence control) and immediately after treatment were tested for fluorescence. Data were evaluated by receiver operating characteristic (ROC) analysis, with Youden’s J statistic used to set a threshold. Pretreatment oral fluid samples with fluorescence responses above the ROC threshold were removed from further analysis (7 of 96 samples). Based on the ROC analyses, oral fluid samples from 78 of 89 pens (87.6%), contained red food coloring, including 43 of 47 (91.5%) pens receiving pig exposure and 35 of 42 (83.3%) pens receiving environmental exposure. Thus, oral fluid samples contain both pig-derived and environmental targets. This methodology provides a safe and quantifiable method to evaluate oral fluid sampling vis-à-vis pen behavior, pen size, sampling protocol, and target distribution in the pen.

## 1. Introduction

Oral fluid specimens are widely used in the swine industry because they are easily collected, contain detectable concentrations of pathogen-specific nucleic acids and antibodies, and provide the means to efficiently establish the exposure status of the group under surveillance [1]. Reflecting their usefulness to pork producers, approximately 2 million polymerase chain reaction (PCR) assays were performed on swine oral fluid samples at six independent veterinary diagnostic laboratories in the U.S. between 2019 and 2022 for influenza A virus (IAV), *Mycoplasma hyopneumoniae*, porcine circovirus type 2 (PCV2), porcine deltacoronavirus (PDCoV), porcine epidemic diarrhea virus (PEDV), porcine reproductive and respiratory syndrome virus (PRRSV), and transmissible gastroenteritis virus (TGEV). Further, the use of oral fluids is widely reported in the refereed literature. Thus, a search in Google Scholar™ using the terms “oral fluid” and “swine” (accessed November 2023) produced 1820 results for the years 2015 to 2023. The majority of these publications addressed various aspects of swine infectious diseases, with oral fluids serving an ancillary role. That is, few publications focused on optimizing or understanding the process of collecting oral fluids. Among the few, White et al. [2] analyzed oral fluid sampling time in the context of pig participation, Kittawornrat and Zimmerman [3] reviewed pig behaviors related to oral fluid collection, and Tarasiuk et al. [4] researched the effect of group size in the context of pig participation in oral fluid sampling. 

Laboratory detection of specific pathogens in oral fluids is widely researched but there is an incomplete understanding of the original source of the target (pig vs. environment). The objective of this study was to evaluate the transfer of a target directly from pigs or indirectly from the environment into pen-based oral fluid samples. In terms of experimental design, this question can be addressed using infectious agents when the work is carried out in research settings. However, in commercial swine herds, the intentional use of infectious agents poses ethical and legal risks to both the collaborating producer and neighboring farms. Under such circumstances, the research is best carried out using noninfectious markers. 

Readily detectable noninfectious tracers have previously been used in animal studies. As reviewed by Algeo et al. [5], tetracycline has been used in oral vaccination studies in coyotes, foxes, raccoons, skunks, feral swine, and other species. Tetracycline binds to calcium ions and is then deposited in teeth and bone (calcified tissues) [6]. In vaccination field studies, animals that consume the vaccine can be identified by ultraviolet visualization of tetracycline in cross-sectioned teeth and/or bone [5]. Similarly, Rhodamine B was used to monitor the uptake of edible oral vaccines in jackrabbits [7], deer [8], and wild boar [9]. Rhodamine B enters the circulatory system and is excreted in urine and feces but it is also sequestered in tissues, hair, and whiskers [10]. Thereafter, the uptake of oral bait vaccines was determined by fluorescent bands of Rhodamine B in hair and whiskers [11]. 

Tetracycline and Rhodamine B are useful for studies in wildlife, but cannot be used in studies involving food animals. Therefore, red food coloring (Red Food Coloring, McCormick and Co., Inc., Hunt Valley, MD, USA) was used as a safe alternative in this research. The commercial product is composed of two food colors (Red No. 40 and Red No. 3) that meet the criteria of the US Federal Food, Drug, and Cosmetic Act for use in food, drugs, and cosmetics, as defined in 21 CFR §74.303 and §74.340. In addition, synthetic food colors are known to fluoresce at specific wavelengths that are measurable using standard laboratory equipment [12]. Thus, fluorescence provided the means to determine whether swine transferred the target (red food coloring) to the pen-based oral fluid sample.

## 2. Materials and Methods

### 2.1. Experimental Design

The study was conducted in commercial production sites using pens holding ~30, ~60, or ~125 pigs, with 32 pens in each size category. Pigs were placed into facilities at 3 weeks of age and were 10–14 weeks of age at the time of the study. In brief, pigs were provided red food coloring suspended in a sucrose solution, a pen-based oral fluid sample was collected, and the sample was tested for fluorescence to determine whether the food coloring was transferred to the sample. To account for naturally occurring fluorescent compounds in the environment, oral fluid samples were also collected one day prior to treatment. Two routes of exposure were evaluated: direct (pig) and indirect (pen floor), in 16 pens for each pen size category. Direct treatment consisted of spraying the solution (~3.5 mL) into the oral cavity of individual restrained pigs (10% of the pigs in the pen). Indirect administration consisted of indirectly exposing the pigs by placing the solution (20 mL) on the floor in the center of the pen. Oral fluid samples were tested for fluorescence using a SpectraMax^®^ i3x Multi-Mode Microplate Reader (Molecular Devices LLC, San Jose, CA, USA). 

### 2.2. Commercial Production Sites

The study was conducted in 3 commercial wean-to-finish production sites (Midwest USA) with curtain-sided, tunnel-ventilated barns built between 2010 and 2018. Pens were fully slatted and equipped with nipple drinkers and standard dry feeders. Barns used in the study were designed to house pigs in pens of ~30, ~60, or ~125 pigs. The study was conducted in 16 pens in each pen size group for each treatment, with pens selected randomly (random.org accessed on 15 May 2023) from all available pens, excluding hospital and recovery pens. 

### 2.3. Oral Fluid Collection and Handling

Oral fluid samples were collected using 100% cotton rope (1.3 cm 3-strand twisted 100% cotton rope, Skydog Rigging Equipment, Lake in the Hills, IL, USA). The rope was placed on the walkway side of the pen ~100 cm from one corner of the pen with the end of the rope suspended at the pigs’ shoulder height. Ropes were hung for 30 min in pens of 30 pigs, 45 min in pens of 60 pigs, and 60 min in pens of 125 pigs. At the end of the collection period, the oral fluid sample was collected by placing the rope in a plastic bag and hand-squeezing to release the fluid. The oral fluid that accumulated in the bottom of the plastic bag was then poured into a 50 mL tube (Southern Labware^®^, Cumming, GA, USA) and transported on dry ice to the laboratory. In the laboratory, oral fluid samples were centrifuged at 3300× *g* for 90 min (J6-HC, Beckman Coulter, Inc., 5350 Lakeview Parkway S drive Indianapolis, IN, USA) at 22 °C, after which the supernatant was transferred to 50 mL tubes (Southern Labware^®^, Cumming, GA, USA) and stored at −20 °C until processed for testing. 

### 2.4. Treatments

#### 2.4.1. Florescence Control Samples

A pretreatment oral fluid sample (background control) was collected from each pen to account for the presence of naturally fluorescing substances in the pen environment, e.g., urine, feces, or feed mycotoxins [13,14,15]. Before collecting the background control oral fluid sample, a mock indirect treatment solution (20 mL) consisting of 50 g of granulated white sugar in 100 mL of distilled water was placed in the approximate center of the pen. 

#### 2.4.2. Direct (Pig) and Indirect (Pen Floor) Treatments

Treatments were performed at the pen level, with each of the 2 treatments randomly assigned to 16 pens (random.org accessed on 15 May 2023) in each pen size category. For both direct and indirect treatment, a stock solution containing a fluorescent food coloring was prepared by mixing 50 g of granulated white sugar in 100 mL of 60 °C distilled deionized water and, after cooling to 20 °C, adding commercial red food coloring (McCormick & Co., Inc., Hunt Valley, MD, USA) in a proportion of 2 parts sugar solution to 1 part food coloring. For direct treatment, a convenience sample of individual pigs (10% of the pen) were restrained, marked for individual identification (Ideal^®^ Prima^®^ Spray-On, Neogen Corp., Lansing, MI, USA), and ~3.5 mL of the stock solution sprayed (Dual Action Spray Bottle, Uline 12575 Uline Drive, Pleasant Prairie, WI, USA) into the buccal cavity. For indirect treatment, 20 mL of the stock solution was poured directly on the floor in the approximate center of the pen to allow pigs free access. 

In pens receiving direct treatment, the entire sampling process was recorded using video cameras (GoPro^®^ 7 cameras, GoPro Inc., San Mateo, CA, USA) strategically placed to clearly record pig interactions with the rope. “Pig–rope contact” was defined as an individually identified pig clearly taking the rope into its mouth. Subsequently, pig–rope contacts were counted at one-minute intervals over the sampling period, i.e., any pig–rope contact during a one-minute interval was counted as “one”. That is, 3 pigs exposed in pens of 30 pigs had a maximum of 90 possible contacts (3 pigs × 30 min). Thus, in pens of 60 and 125 pigs, the maximum possible contacts were 270 (6 pigs × 45 min) and 720 (12 pigs × 60 min), respectively. Pig participation at the pen level was defined as the number of marked pigs that had one or more pig–rope contacts during the sampling time. Maximum values for pig participation were 3, 6, and 12 in the direct treatments for pen size categories of 30, 60, and 125, respectively. 

#### 2.4.3. Relative Fluorescence Units (RFUs)

Pen-based oral fluid samples were tested for fluorescence using the SpectraMax^®^ i3x Multi-Mode Microplate Reader (Molecular Devices LLC) and control software (SoftMax^®^ Pro 6.5 Molecular Devices LLC), with the response reported in relative fluorescence units (RFUs). Initially, the optimized excitation and emission wavelengths of the red food coloring were established as 530 nm and 570 nm, respectively, using the control software spectral optimization function. For testing, oral fluid samples were thawed overnight at 4 °C and centrifuged at 3300× *g* for 10 min (J6-HC, Beckman Coulter, Inc., Indianapolis, IN, USA) at 22 °C. Thereafter, the supernatant was transferred to a 50 mL tube, vortexed, and tested in duplicate by pipetting 100 µL into one well in each of two 96-well black-walled and clear bottom plates (Thermo Fisher Scientific™ Nunc, Waltham, MA, USA). Concurrently, a standard curve based on the stock solution (Section 2.4.2) was run in duplicate on each plate (blank, 1:100 and then 2-fold dilutions through 1:102,400). To account for background and for the effect of the oral fluid matrix on fluorescence readings, the diluent for the standard curve dilutions was created by pooling 0.5 mL of the pretreatment oral fluid samples collected from each pen (*n* = 32) in a pen size category. Thus, 3 separate and specific standard curves were created, i.e., one for each pen size category.

### 2.5. Data Analysis

All analyses were performed using R software v4.3.1 [16] and RStudio v2023.03.0. Prior to analyzing the data, the mean RFU of each pen-based oral fluid sample (Section 2.4) was calculated from technical duplicates and log transformed (lnRFU). Mean lnRFU and 95% confidence intervals were calculated for each treatment and pen size combination using the summarise() function of the R dplyr() package v1.1.3 [17]. Thereafter, the lnRFU threshold value that optimally differentiated between pre- and post-treatment oral fluid sample lnRFU responses (Youden’s J statistic) was determined by receiver operating characteristic (ROC) analysis using the pROC package v1.17.0.1 [18]. Six ROC analyses were performed, i.e., one for each combination of pen size (*n* = 3) and treatment (*n* = 2). 

To maximize the specificity of the estimates, pen samples (*n* = 7) with pretreatment background lnRFU responses above the lnRFU threshold (plus a 0.001 tolerance) were excluded from all subsequent analyses. For the remaining eligible samples (*n* = 89), post-treatment mean lnRFUs and 95% confidence intervals were recalculated for each combination of treatment and pen size. Pen samples (*n* = 43) with post-treatment lnRFU values greater than the threshold (plus a 0.001 threshold) were retained for the remainder of the analysis. Thereafter, lnRFU responses were calculated as a percent (%) of the exposure stock solution (Section 2.4.3) based on a linear calibration using the standard curve run on each plate and the inverse.predict function of R chemCal() package v0.2.3 [19]. Pen size category mean lnRFU values for the direct and indirect treatment groups were compared by analysis of variance (ANOVA) followed by pairwise comparisons using R-base functions aov() and TukeyHSD() when the overall F-statistic was significant. This analysis was then repeated for the mean concentration of red food coloring (percent stock solution). Post hoc tests *p*-values were adjusted using Tukey’s method to control a familywise error rate of 0.05. The relationship between the total number of pig–rope contacts (count data derived from the video recordings) and post-treatment oral fluid sample lnRFU responses was evaluated using a linear regression fit using the geom_smooth function with method = lm in ggplot2 [20]. The relationship between pen-level pig participation and post-treatment oral fluid sample lnRFU responses was also evaluated using a linear fit.

## 3. Results

The pre- and post-treatment lnRFU responses (mean, 95% CI) and ROC response thresholds by pen size and treatment route are presented in a stepwise fashion in Table 1 (Steps 1, 2). 

The ROC analyses produced six independent lnRFU threshold values by pen size category and route of treatment (Table 1, Step 3). The pretreatment pen sample lnRFU exceeded the ROC threshold in 7 (7.35%) of the 96 pens in the study. Therefore, these pens were excluded from subsequent analyses due to excessive background fluorescence (Table 1, Step 3a). 

Among the remaining 89 pens, ROC analysis of post-treatment lnRFU data showed that food coloring was detected in 43 of 47 (91.5%) post-treatment samples from pens receiving direct treatment and 35 of 42 (83.3%) pens receiving indirect treatment (Table 1, Step 3b). Among the 43 direct treatment pens in which food coloring was detected, ANOVA of the post-treatment lnRFU data showed a difference in response (*p* < 0.001) by pen size. Post hoc pairwise comparisons between direct treatment groups detected no difference in mean lnRFU in samples from pens of 30 vs. 60 pigs (Tukey’s test, *p* = 0.986), but comparisons of 30 vs. 125 pigs (Tukey’s test *p* < 0.001) and 60 vs. 125 pigs (Tukey’s test *p* < 0.001) detected significant differences. In contrast, no difference in response was detected among pen sizes for pens receiving indirect treatment (ANOVA, *p* = 0.171). 

Among the 78 pen samples reported in Table 1, Step 3b, the concentration of red food coloring was calculated as a percent (%) of the stock solution based on an analysis of the standard curves run on each plate. In pens receiving direct treatment, the mean concentrations (95% CI) were 0.0047% (0.0034, 0.0060), 0.0049% (0.0038, 0.0060), and 0.046% (0.031, 0.062) for pens sizes of 30, 60, and 125 pigs, respectively. In pens receiving indirect treatment, the mean concentrations (95% CI) were 0.0036% (0.0028, 0.0045), 0.0037% (0.0032, 0.0042), and 0.0033% (0.0029, 0.0038) in pen size categories of 30, 60, and 125 pigs, respectively. Among pens receiving direct treatment, a significant difference in the concentration of red food coloring was found among pen sizes (ANOVA, *p* < 0.001). Post hoc pairwise comparisons detected no difference in pens of 30 vs. 60 pigs (Tukey’s test, *p* = 0.999), but a significant difference was found in pens of 30 vs. 125 pigs (Tukey’s test, *p* < 0.001) and 60 vs. 125 pigs (Tukey’s test, *p* < 0.001). No difference in the concentration of red food coloring was detected among pen sizes for pens receiving indirect treatment (ANOVA, *p* = 0.628).

**Figure 1:**
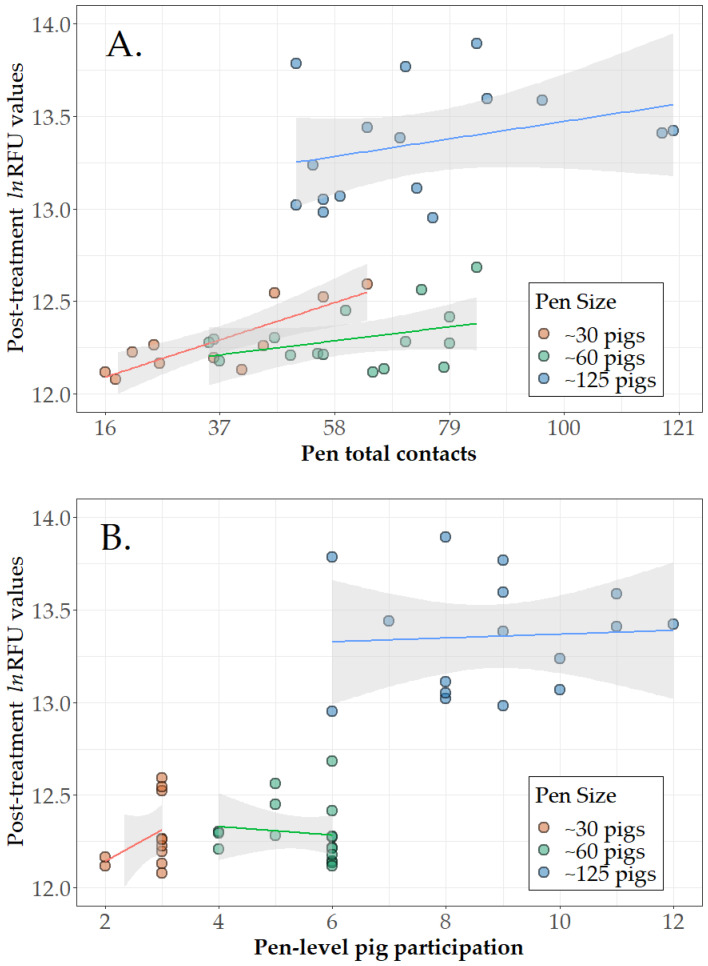
Direct treatment results. Scatterplots of post-treatment relative fluorescent units (ln(RFU)) in pen-based oral fluids versus (**A**) number of pig–rope contacts by individually marked pigs and (**B**) number of marked pigs that participated with fitted linear regression lines and 95% confidence intervals. Figure 1 is based on 43 pens receiving direct treatment and classified as positive in the ROC analyses (Table 1 Step 3). Note that 10% of pigs per pen were individually treated and marked, i.e., 3, 6, and 12 pigs in pens of ~30, ~60, and ~125 pigs, respectively. Oral fluid sampling time varied by pen size, i.e., 30 min in pens of ~30 pigs, 45 min in pens of ~60 pigs, and 60 min in pens of ~125 pigs.

Among the 43 direct treatment pens (Table 1, Step 3b), pen-level pig participation (number of marked pigs contacting the rope at least once in the pen) and pen total contacts (number of one-minute intervals in which any marked pig in the pen contacted with a rope) are presented in Figure 1A,B. By pen size, mean pen-level pig participation was 2.8 of 3 pigs (2.6, 3.0), 5.4 of 6 pigs (5.0, 5.9), and 8.8 of 12 (7.9, 9.7) for pens of 30, 60, and 125 pigs, respectively. The mean number of pig–rope contacts was 35.9 (95% CI 25.1, 46.7) out of a possible total of 90 for pens of 30, 60.8 (52.1, 69.6) out of a possible total of 270 for pens of 60, and 74.1 (62.4, 85.8) out of a possible total of 720 for pens of 125 pigs. Linear regression analysis (lm() function of base R) showed that the number of contact events had a significant linear relationship to a pen’s RFU response for pens of 30 pigs, i.e., on average, each contact event produced an increase of 0.0096 in lnRFU. The slopes were not significant for pens of 60 (*p* = 0.154) or 125 (*p* = 0.226). Slopes were not significant for pen-level pig participation for any of the pen sizes (*p* > 0.257).

## 4. Discussion

In humans and animals, hormones, proteins, antibodies, viruses, and other bioelements in the circulatory system enter the buccal cavity via the salivary glands, mucus membranes, and gingival crevices by ultrafiltration, transudation, passive diffusion, and/or active transport [21]. This occurs as a highly dynamic process. For example, Brill and Krasse [22] demonstrated the transfer of fluorescein dye from the circulatory system into gingival crevicular fluid within 30 s of intravenous injection in dogs. Similar results were observed in humans who consumed capsules containing fluorescein dye [23] and humans intravenously injected with albumin labeled with iodine-131 [24]. In the context of infectious diseases, Madonia et al. [25] demonstrated the transfer of Coxsackie B-1 virus from the circulatory system into oral fluids by injecting the virus into a marginal ear vein of rabbits and detecting the virus in oral fluids within two minutes. In human medicine, human immunodeficiency virus (HIV) [26] and HIV antibody [27] were reported in oral fluids collected from patients with immunodeficiency syndrome (AIDS) or AIDS-related complex (ARC). Moving quickly, in 1995, the FDA approved an HIV antibody testing system based on oral fluids rather than blood [28]. 

Ongoing research has produced a number of practical and widely used applications for oral fluids, e.g., assessment of drug use [29] and oral fluid-based testing of human populations for HIV [30], measles [31], hepatitis A [32], severe acute respiratory syndrome coronavirus 2 (SARS-CoV-2) testing [33], and others. In pigs, the first diagnostic application of oral fluids was reported by Prickett et al. in 2008 [34]. Specifically, PRRSV RNA and PRRSV antibodies were detected in oral fluids collected from pigs inoculated with the virus under experimental conditions. Since then, an additional 16 bacterial and 25 viral pathogens of swine have been detected in oral fluids under experimental and field conditions [35]. Because the approach is both convenient and effective, oral-fluid-based testing is widely used by swine producers and veterinarians for a variety of pathogens [36]. In 2022, 460,150 PCR assays were performed on oral fluids for seven major pathogens of swine (IAV, *Mycoplasma hyopneumoniae*, PCV2, PDCoV, PEDV, PRRSV, and TGEV) at five diagnostic laboratories (Iowa, Kansas, Ohio, Minnesota, and South Dakota) accredited by the American Association of Veterinary Laboratory Diagnosticians [37].

While the detection of nucleic acids in oral fluids may reflect a pathogen’s systemic distribution, it may also represent local replication in oropharyngeal tissues. As reviewed by Lilja et al. [38], in humans, bacterial and viral pathogens that replicate in tonsils include Epstein–Barr virus, adenovirus, influenza virus, parainfluenza virus, coxsackie A virus, herpes virus, rhino virus, coronavirus, *Streptococcus pyogenes*, *Neisseria gonorrhoeae*, *Corynebacterium diphtheriae*, and *Borrelia vincentii*. Similarly, in pigs, a variety of bacterial and viral pathogens replicate in and/or colonize tonsils including *Salmonella typhimurium*, *Salmonella choleraesuis*, *Streptococcus suis*, African swine fever virus, classical swine fever virus, PCV2, PRRSV, foot-and-mouth disease virus, and others [39]. As reviewed by Munguía-Ramírez et al. [35], all of these pathogens have been detected by PCR in swine oral fluids.

Thus, diagnostic targets performed on oral fluid may represent systemic infection or replication in buccal tissues. In swine and other animals, diagnostic targets in oral fluid samples may also reflect their presence in the environment [40]. Normal swine behavior includes rooting, biting, smelling, and tasting the environment. Diagnostic targets encountered in the environment during this exploratory process may be retained in the oral cavity, deposited in the oral fluid sample, and detected by testing [3]. The diagnostic relevance of this process was first illustrated by Johnson et al. [41] who showed that the source of PRRSV antibody detected in oral fluid samples from PRRSV negative pigs was porcine dry-sprayed plasma in the feed provided to the animals. The consistency and repeatability of the transfer of targets from the environment into oral fluid samples is exemplified by the routine detection of enteric pathogens in oral fluids, e.g., *Brachyspira* ssp. [42], *Lawsonia intracellularis* [42], PDCoV [43], PEDV [44,45], and TGEV [46].

Despite the strength of the empirical evidence, the detection of environmental targets in oral fluid samples has not been proven under controlled conditions. To address this deficiency, we documented the transfer of a tracer directly from pigs or indirectly from the pigs’ environment into pen-based oral fluid samples in active swine farms over a range of pen sizes. To address the ethical and legal risks posed by the use of infectious agents in commercial farms, and yet precisely define exposure conditions in the field, an inert tracer was used. Broadly speaking, the use of chemical tracers in field research is well-documented [47]. Examples in veterinary medicine include the use of tetracycline to document oral rabies vaccine uptake in racoons [48] and Rhodamine B to document classical swine fever virus oral vaccine uptake in wild boar [9,49]. In this study, the requirements for the tracer included safety, availability, low cost, ease of detection, and suitability for use in food animals. None of the previously described tracers meet these qualifications. In contrast, the food colorings used in this study (Red No. 3 and Red No. 40) are approved by the U.S. FDA for use in foods, drugs, and cosmetics and are widely available in food markets at a reasonable cost. Further, under proper storage conditions, i.e., cool, dry, and shielded from light, the manufacturer ascribes a shelf life of 1440 days [50]. 

This study made use of the fact that food colorings fluoresce at specific wavelengths that may be detected with standard laboratory equipment [12]. The optimum fluorescence for the red food coloring used in this study was achieved at 530 nm and 570 nm for excitation and emission, respectively. The possible presence of naturally fluorescent compounds in the livestock environment (background fluorescence), e.g., urine, mycotoxins, and feces [13,14,15], was accounted for by including pretreatment oral fluid sample RFU responses in the ROC analysis. In the experimental design, the transfer of a target from pigs into oral fluids was observed from direct exposure of 10% of pigs in the pen or from indirect exposure of 20 mL tracer solution on the floor of the pen. It is worth noting that when the amount of dye introduced into the environment was held constant, there was no significant difference in the mean lnRFU or concentration as percent of the stock solution for oral fluids samples collected across different pens sizes. This suggests that, as pen size and pig numbers increase, diagnostic targets in the environment are nevertheless consistently deposited in oral fluid samples. As expected, when the amount of dye introduced into pens was increased by direct exposure of a higher number of animals, the mean lnRFU in oral fluid samples increased. This supports the idea that higher burdens of biologic targets are likely to increase the concentration, and therefore likelihood of detection, of the target in oral fluid samples. Variations in the experimental design using food coloring (or other safe tracers) could be used to address other questions regarding behavioral and mechanical aspects of swine oral fluid sampling, e.g., holding sampling time and pen size constant and changing number of pigs exposed. Regardless, the present study demonstrated the repeatability of the transfer of targets into oral fluid samples and described a new tool for further epidemiological studies. 

## 5. Conclusions

The objective of this study was to evaluate the repeatability of the transfer of a target directly from pigs or indirectly from the environment into pen-based oral fluid samples over a range of pen sizes in commercial swine farms. Red food coloring was used as the target because of its safety, availability, and detectability (fluorescence) profile. Direct exposure of ~10% of the pigs in a pen resulted in detection of the fluorescent marker in 73.3% (11 of 15) of the pen-based oral fluid samples from pens of ~30 pigs and 100% of samples from pens of ~60 and ~125 pigs (16 of 16 pens for each). For environmental targets, the marker was detected in 85.7% (12 of 14 pens), 92.9% (13 of 14 pens), and 71.4% (10 of 14 pens) of oral fluid samples from pens holding ~30, ~60, and ~125 pigs, respectively. Thus, targets to which the pigs have access are routinely transferred into oral fluid samples. Although the surrogate target used in this study was intentionally administered to pigs or placed in the environment, the results provided a rational explanation for the routine detection of swine enteric pathogens, i.e., pathogens not normally in the upper respiratory tract or oropharynx, in pen-based oral fluid samples. In conclusion, on commercial swine farms, the use of red food coloring provided a safe, inexpensive, specific, and analytically sensitive method for documenting the transfer of the target directly from pigs or indirectly from the environment into pen-based oral fluid samples.

## Figures and Tables

**Table 1:** Detection of fluorescence in pen-based oral fluid samples reported as log-transformed relative fluorescence units (ln(RFU)) following exposure to food coloring by route (direct, indirect) and pen size (30, 60, or 125 pigs per pen).

	Step-Wise Analysis	Pen Size (Sampling Time)		
		30 Pigs per Pen (30 min)	60 Pigs per Pen (45 min)	125 Pigs per Pen (60 min)
Direct treatment ^2^	1. Pre-treatment lnRFU means based on 16 pens per pen size (95% CI)	11.86 ^1^ (11.78, 11.93)	11.87 ^1^ (11.77, 11.96)	11.78 ^1^ (11.73, 11.82)
	2. Post-treatment lnRFU means based on 16 pens per pen size (95% CI)	12.17 (12.05, 12.30)	12.30 (12.21, 12.38)	13.36 (13.19, 13.52)
	3. ROC threshold (lnRFU) ^3^ a. Pre-treatment pen samples > ROC threshold ^4^ b. Post-treatment pen samples > ROC threshold (lnRFU mean, 95% CI) ^5^	12.06 1 of 16 pens 11 of 15 pens 12.28 ^a^ (12.16, 12.40)	12.10 0 of 16 pens 16 of 16 pens 12.30 ^a^ (12.21, 12.38)	12.45 0 of 16 pens 16 of 16 pens 13.36 ^b^ (13.19, 13.52)
Indirect treatment ^2^	1. Pre-treatment means based on 16 pens for each pen size (95% CI)	11.83 (11.76, 11.90)	11.78 (11.67, 11.89)	11.76 (11.67, 11.84)
	2. Post-treatment means based on 16 pens for each pen size (95% CI)	12.13 (12.02, 12.24)	12.17 (12.08, 12.25)	12.04 (11.94, 12.14)
	3. ROC threshold (lnRFU) ^3^ a. Pre-treatment pen samples > ROC threshold ^4^ b. Post-treatment pen samples > ROC threshold ^5^ (lnRFU mean, 95% CI)	11.96 2 of 16 pens 12 of 14 pens 12.16 ^a^ (12.06, 12.26)	11.99 2 of 16 pens 13 of 14 pens 12.18 ^a^ (12.12, 12.25)	11.88 2 of 16 pens 10 of 14 pens 12.11 ^a^ (12.04, 12.17)

^1^ Fluorescence in pen-based oral fluid samples quantified using a multi-mode microplate reader (SpectraMax^®^ i3x Multi-Mode Microplate Reader, Molecular Devices LLC, San Jose, CA, USA) and control software (SoftMax^®^ Pro 6.5 Molecular Devices LLC), read as relative fluorescent units, and then log transformed (ln(RFU)) for analysis and reporting. ^2^ Direct treatment: 10% of pigs in pen individually restrained and stock solution (~3.5 mL) sprayed into buccal cavity. Indirect treatment: stock solution (20 mL) poured on floor in center of pen. ^3^ Threshold derived from receiver operating characteristics analysis (R package pROC v1.17.0.1 [18]) of pre- and post-treatment ln(RFU) results. A separate ROC was performed for each combination of (treatment × pen size). ^4^ Pretreatment oral fluid samples collected from each pen (16 per pen size category) one day prior to treatment. Pens with pretreatment samples > ROC threshold were disqualified from further analysis on the basis of excess background fluorescence. ^5^ Post-treatment oral fluid samples collected immediately after treatment. Estimates in Step 3b do not include pens eliminated in Step 3a. ^a,b^ Tukey’s test *p* < 0.001.

## Data Availability

The data that support the findings of this study are available on request from the corresponding author.
